# Predicting chemoinsensitivity in breast cancer with ’omics/digital pathology data fusion

**DOI:** 10.1098/rsos.140501

**Published:** 2016-02-10

**Authors:** Richard S. Savage, Yinyin Yuan

**Affiliations:** 1 Systems Biology Centre, University of Warwick, Warwick, UK; 2 Warwick Medical School, University of Warwick, Warwick, UK; 3 Division of Molecular Pathology, Centre for Evolution and Cancer, The Institute of Cancer Research, London, UK

**Keywords:** breast cancer, data integration, Bayesian

## Abstract

Predicting response to treatment and disease-specific deaths are key tasks in cancer research yet there is a lack of methodologies to achieve these. Large-scale ’omics and digital pathology technologies have led to the need for effective statistical methods for data fusion to extract the most useful patterns from these diverse data types. We present *FusionGP*, a method for combining heterogeneous data types designed specifically for predicting outcome of treatment and disease. *FusionGP* is a Gaussian process model that includes a generalization of feature selection for biomarker discovery, allowing for simultaneous, sparse feature selection across multiple data types. Importantly, it can accommodate highly nonlinear structure in the data, and automatically infers the optimal contribution from each input data type. *FusionGP* compares favourably to several popular classification methods, including the Random Forest classifier, a stepwise logistic regression model and the Support Vector Machine on single data types. By combining gene expression, copy number alteration and digital pathology image data in 119 estrogen receptor (ER)-negative and 345 ER-positive breast tumours, we aim to predict two important clinical outcomes: death and chemoinsensitivity. While gene expression data give the best predictive performance in the majority of cases, the digital pathology data are much better for predicting death in ER cases. Thus, *FusionGP* is a new tool for selecting informative features from heterogeneous data types and predicting treatment response and prognosis.

## 1. Introduction

Post-genomic molecular biology is revolutionizing the study of cancer [2,1]. New measurement technologies are giving us fresh insights into the molecular and genetic mechanisms underlying the disease, and modelling these data gives us ways to predict the likely progression and outcome of disease in a given patient [3,4]. By identifying informative markers related to critical events, there is unprecedented potential for both the development of new prognostic/diagnostic tests, and also for furthering our understanding of cancer's key driving molecular and genetic mechanisms.

The digitisation of high-quality tumour pathology images now gives us a complementary perspective at the point of diagnosis [5]. Tumour cells can be identified in these images computationally and the resulting data on cell morphology and spatial distribution used as an additional source of information in analysing a given cancer case. We therefore have access to multiple data types for a given cancer patient. The most effective prediction of clinical outcomes then requires that we develop statistical algorithms that are able to effectively combine these diverse data types. This presents several challenges which we aim to address in this paper.

(i) *Challenge* 1: *outcome prediction.* Given a training set of measurements, we wish to predict the likely outcomes of treatment or prognosis for a new patient for whom we have analogous measurements. This can inform clinicians of the likely disease course and hence help design efficient treatment strategy.
(ii) *Challenge* 2: *biomarker discovery.* The available data are often high-dimensional. By identifying in a principled way which features are informative by allowing for the possibility of *sparse solutions*, we identify a small subset of the features are informative about clinical outcome.
(iii) *Challenge* 3: *effective integration of multiple data types.* To get the best possible results, we should include all relevant information and do so in a principled way. In particular, different types of data offer complementary perspectives on a given disease. We, therefore, wish to generalize the notion of feature selection to include multiple, possibly heterogeneous data types.

There have been a number of previous attempts to address various of these challenges, for example, Futschik *et al.* [6], Nevins *et al.* [7], Pittman *et al.* [8], Stephenson *et al.* [9], Gevaert *et al.* [10], Boulesteix *et al.* [11], Daemen *et al.* [12], Obulkasim *et al.* [13]. However, the field as yet lacks a comprehensive framework that is suitable for combining data from the types of highly heterogeneous biomedical data that are being generated in increasingly large volumes. To address this, we adopt a statistical modelling approach. Such a model must be able to handle high-dimensional, noisy data in a principled way and must be capable of learning a wide range of possible structure. Non-parametric Bayesian models provide just such a framework. By learning a level of structure appropriate to the data, they naturally encode a kind of Occam's razor, and their Bayesian nature lends itself naturally to the inclusion of multiple sources of data and/or prior knowledge. We have previously developed Bayesian non-parametric models for data integration in an unsupervised setting, e.g. Savage *et al.* [14], Yuan *et al.* [15], Kirk *et al.* [16], but there is much work still to be done in the supervised setting.

## 2. Material and methods

We present *FusionGP*, a Bayesian non-parametric method for integrating multiple data types, to perform classification and sparse biomarker discovery. The relationship between input features and outcome is modelled via a set of unknown latent functions (one per data type) that are constrained via a set of Gaussian process (GP) priors. The input features for each data type are selected via a slab-and-spike prior, where the probability of a feature being *switched on* is inferred as part of the inference process, allowing for sparse feature selection in a given data type, where the data support it.

### 2.1 Gaussian processes

We model the relationship between each data type and the target values via (unknown) latent functions. We assume the latent functions to be realizations of a zero-mean GP [17], noting that this approach has a successful track record in modelling molecular data [18–20].

For convenience, we adopt a compact notation for the latent function for the *d*th data type, ***f***_***d***_. Given this, we define the following GP prior over the space of unknown functions for the *d*th data type:

2.1 $$P(\;{\text{f}}_{\text{d}})={\left(2\pi \right)}^{-n/2}|K{|}^{-1/2}\text{exp}\left(-\frac{1}{2}{{\text{f}}_{\text{d}}}^{T}{\left(K\right)}^{-1}{\text{f}}_{\text{d}}\right).$$

For *n* data points, and where *K* is the *N*×*N* covariance matrix that defines the GP.

### 2.2 Feature selection

For GP models, the features contribute via the covariance function. To allow feature selection, we define additional indicator parameters, *I*_*jd*_ for the *j*th feature in the *d*th data type . When *I*_*jd*_=1, the *j*th feature is *switched on* and behaves as usual. When *I*_*jd*_=0, the feature is excluded from the analysis. In molecular data, we expect that in general the number of features that are informative may be relatively small. We, therefore, apply a sparsity prior to the *I*_*jd*_, to encode this knowledge:

2.2 $$P(I_{jd})\propto 2^{-a_{d}}w_{d}^{a_{d}}{\left(1-w_{d}\right)}^{J_{d}-a_{d}},$$

where *a*_*d*_=*Σ*_*j*_*I*_*jd*_, the number of features switched on in data type *d* and *J*_*d*_ is the total number of features in data type *d*.

This is, therefore, a product of Bernoulli distributions (one per indicator parameter), with an additional factor of 2^−*a*_*d*_^ which penalizes individual features being switched on. This equation is, therefore, the joint distribution for all *I*_*jd*_ for a given data type.

The hyperparameters *w*_*d*_ are inferred and allow the model to determine the appropriate level of sparsity, given the data. We apply a binomial prior on *w*_*d*_, using this to encode our prior belief that at least a small number of the features will be informative. We also enforce a hard prior of *w*_*d*_≤0.5, because we do not wish the prior itself to favour switching features on:

2.3 w∼β(10,10).

### 2.3 Covariance function

One of the great advantages of GP models is that we have access to a wide range of covariance functions, all using the same overall model framework. For the synthetic data analyses in this paper, we use four different covariance functions:

2.4 $$\begin{array}{rl} K_{\mathrm{linear}}(x_{i},x_{j}) & =A_{0}+{\text{x}}_{\text{i}}.{\text{x}}_{\text{j}}, \end{array}$$

2.5 $$\begin{array}{rl} K_{\mathrm{SE}}(x_{i},x_{j}) & =\exp\left(-\frac{r^{2}}{2l^{2}}\right), \end{array}$$

2.6 $$\begin{array}{rl} K_{\mathrm{Matern}}(x_{i},x_{j}) & =\left(1+\frac{\sqrt{3}r}{l}\right)\exp\left(-\frac{\sqrt{3}r}{l}\right) \end{array}$$

2.7 $$\begin{array}{rl} \text{and}\;K_{\mathrm{sum}}(x_{i},x_{j}) & =K_{\mathrm{linear}}(x_{i},x_{j})+K_{\mathrm{SE}}(x_{i},x_{j}), \end{array}$$

where ‘SE’ stands for ‘square exponential’ and *r*=|***x***_***i***_−***x***_***j***_|.

For simplicity, all analyses presented in this paper use the same covariance function for data types in said analysis. This is chosen for convenience—*FusionGP* does not require this. *FusionGP* uses the GPML Matlab library [21], which means that it can be run with a wide range of different covariance functions. We are grateful to the GPML authors for their provision of this library and would recommend it highly to anyone working with GPs in Matlab.

### 2.4 Model specification

To model binary outcomes, we choose the probit function [22]:

2.8 $$P(D|{\text{f}}_{\text{d}})∼\mathrm{probit}.$$

We model each data type using an (unknown) latent function, that relates the input features ***x*** to the outcome of interest, *y*, via the probit likelihood. We wish these latent functions to be highly flexible so that they can capture the complex biological structure underlying the data. We therefore choose to draw the latent functions from zero-mean GPs:

2.9 $${\text{f}}_{\text{d}}∼\mathrm{GP}(0,K_{d}).$$

To include information from multiple data sources, we use the approach of Girolami & Zhong [23]. In this approach, we assume that the datasets are conditionally independent of one another given the target values. This results in a model that is easy to work with and is nevertheless extremely powerful. The overall latent function is therefore a linear combination of the individual latent functions:

2.10 $$\text{f}=\sum_{d}A_{d}{\text{f}}_{\text{d}},$$

where *A*_*d*_ is the scaling parameter for the *d*th latent function. We note that this model has the great merit of scaling well with the number of datasets. For example, the number of covariance parameters is proportional to the number of datasets.

**Figure 1.**
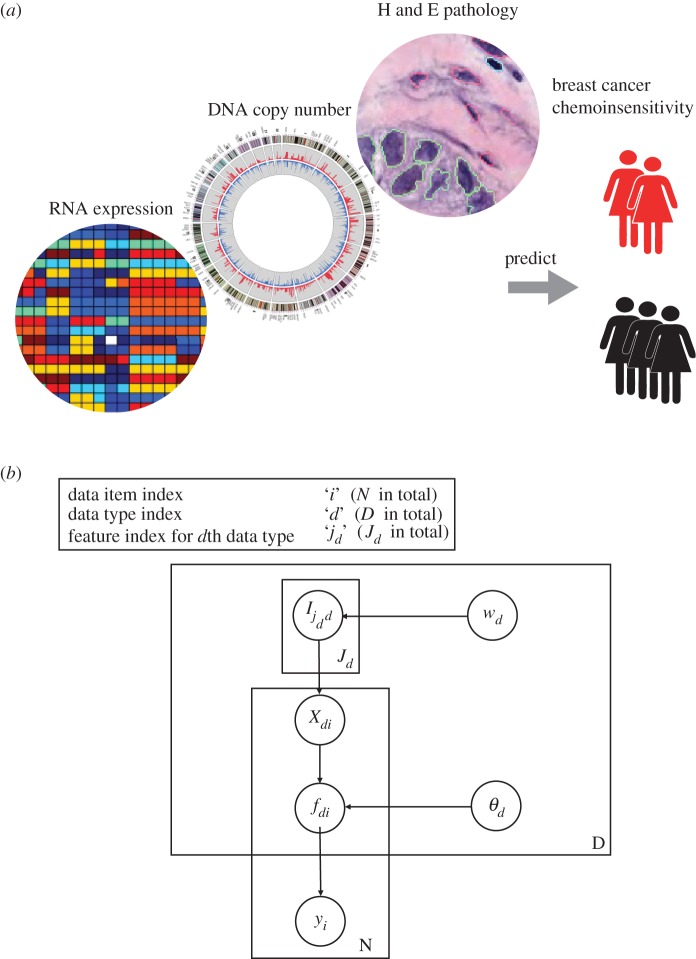
(*a*)The aim of *FusionGP* is to predict clinical outcome such as chemoinsensitivity by integrative analysis of multiple data types. (*b*) Graphical model for *FusionGP*. *y*_*i*_ are the target labels. *f*_*di*_ is the latent function value for the *i*th data item in the *d*th data type. *θ*_*d*_ are the hyperparameters that define the covariance function for the *d*th data type. *X*_*di*_ is the data value for the *i*th data item in the *d*th data type. *I*_*j*_*d*_*d*_ is the feature selection indicator parameter for the *j*_*d*_**th feature in the *d*th data type. *w*_*d*_ are the hyperparameters that encode sparsity in the feature selection.

The features in each data type are assigned indicator parameters, *I*_*jd*_, subject to the sparsity priors discussed in the above section on feature selection. The covariance matrix for a given data type is computed using only the features for which *I*_*jd*_=1. This, therefore, encodes sparse feature selection into the *FusionGP* model. A graphical model representation of the *FusionGP* model is shown in figure 1.

### 2.5 Inference

Owing to the form of the likelihood, this model does not have a closed form. We, therefore, use Markov chain Monte Carlo (MCMC) to perform inference. The challenge in doing this is one of computation time. MCMC analysis typically requires at least *O*(10^4^−10^5^) evaluations of the likelihood, and inference with GPs requires Cholesky decompositions of the covariance matrix for each evaluation, which scale as the cube of the number of data items. For types of data considered in this paper, the number of data items will be tractable.

In the context of GPs, Cholesky decompositions tend to become challenging to use for more than *O*(10^4^) data items. Given that this method uses MCMC (rather than parameter optimization), we suspect that for more than a few thousand data items, the proposal model will be sufficiently slow as to be intractable. However, there are now many effective ways to scale GPs to larger numbers of data items, so this would probably provide a plausible way forward in such cases.

We note that the Cholesky decomposition has no dependence on the number of features. For the covariance functions considered in this paper, computing the covariance matrix is linear in the number of features, and will typically not be the dominant step in the algorithm. *FusionGP*, therefore, scales extremely well with the both the number of features in each data type, and also the total number of features across all data types.

We sample the logarithm of the covariance hyperparameters (*w*_*d*_ and *A*_*d*_) using a Metropolis–Hastings algorithm with Gaussian proposal distribution, the variance of which we tune using samples from an initial burn-in period of the MCMC chain. We choose to work with the log-parameters for convenience as the parameters cannot take negative values.

When using GPs, we marginalize over the (unknown) latent function. Because in *FusionGP* the likelihood is not closed form, we do this as part of the MCMC algorithm. Specifically, we define a set of parameters that are the latent function values at the training points. We sample these using a Metropolis–Hastings algorithm with Gaussian proposal distribution, with variance again tuned using samples from an initial burn-in period of the MCMC chain. The latent function values are then effectively marginalized as part of the MCMC sampling process. We note that there are more sophisticated sampling schemes that one could use here, such as elliptical slice sampling [24]. We found a simpler scheme adequate for the results presented in this paper, but future improvements can certainly be made to the sampling scheme.

### 2.6 Markov chain Monte Carlo performance

For each analysis in this paper, we run five MCMC chains and combine the results, after removing the first 50% as burn-in and sparse-sampling the chains by a factor of 10. Each chain is run for 48 h in the University of Warwick's High Performance Computing cluster. The results presented in this paper are, therefore, produced using a fixed total run-time.

We note that MCMC convergence can be challenging for models dealign with high-dimensional feature selection, and *FusionGP* is no exception. We assess the convergence of our analyses by looking by eye at a range of one-dimensional marginal posteriors (histograms), comparing the equivalent plot for each of the chains (which should give the same distribution if the chains are converged). This could be further improved in future by taking a more in-depth statistical approach, but our practical experience is that this approach works well in practice.

## 3. Examples

We validate *FusionGP* on both synthetic and real breast cancer data. The synthetic data are generated to provide a rigorous test of *FusionGP's* performance where the ground truth is known, and to facilitate comparisons with existing methods. The breast cancer dataset is taken from the recently published METABRIC study of over 2000 patients [25].

### 3.1 Synthetic data

We generate two synthetic data types, each with the same underlying signal, and different noise realizations drawn from the same Gaussian distribution. These data are designed to represent the case where we have a significant number of features, of which a relatively small proportion are informative.

Each data type contains 500 items (250 *outcome*=*true* and 250 *outcome*=*false* cases). There are 600 features, 100 of which contain signal and the remaining 500 of which contain only noise. Each signal feature is generated by taking the known target values and adding Gaussian noise, whose variance is fixed for a given data type. The noise features are generated solely by drawing from the same Gaussian noise distribution, i.e. without any signal.

### 3.2 METABRIC breast cancer data

METABRIC is a large-scale study of the genomic and transcriptomic landscape of breast cancer [25]. The METABRIC data contains copy number, gene expression, histopathological haematoxylin and eosin (H&E) images, and clinical information for over 2000 patients. H&E stained images of tumour slides are potentially highly complementary to the molecular data types. The METABRIC images have been quantitatively analysed by Yuan *et al.* [5]. Nuclear morphological features of different types of cells including cancer, lymphocytes and stromal cells which include fibroblasts and endothelial cells were quantitatively measured. These features include topological and image moment features to measure elongation, size, texture, etc., yielding 100 features in total. Median, variance and skewness were used to characterize the distribution of nuclear features in each tumour. As a result, we obtained imaging data for each patient tumour where each of the morphological features was summarized by median, variance or skewness, totalling 900 features.

We considered two clinical outcomes: death and chemoinsensitivity. All are defined as binary outcomes for simplicity of modelling. This will result in some information loss on the case of death, which would be more properly modelled as a (censored) survival time. However, this choice allows us to focus in paper more on other aspects of the model. We, therefore, considered only samples from patients who have either survived for 10 years, or who are confirmed to have died prior to that point. This leaves us with 464 samples for which we have copy number, gene expression and image feature data. Of these 464 samples, 119 were estrogen receptor (ER) negative (ER−) and 345 were ER positive (ER+). In addition to analysing the whole set of samples, we also considered the ER− and ER+ subsets, as these subsets are biologically distinct disease from one another. We, therefore, define the clinical outcomes as follows.

— *Dea*th. The patient died of breast cancer within 10 years of diagnosis with breast cancer.
— *Chemoinsensitivity*. The patient died (as above) and had been treated with chemotherapy.

## 4. Results

### 4.1 Results on synthetic data

Tables 1 and 2 show the results of *FusionGP* analyses of the synthetic data. Table 2 gives ‘oracle’ results, where only ‘signal’ features are used in the analysis. Table 1 gives the results for the case where the correct features are not known, so the algorithm must use feature selection. Comparing the two sets of results, we can see that while the presence of noise features has an adverse impact on the outcome prediction area under curve (AUC), we are still able to make useful predictions even when there are five times as much noise as informative features. The biomarker AUC values show that correctly identifying the informative ‘biomarker’ features is more challenging than simply making predictions. This may be an indication as to one reason why biomarker discovery in genomic cancer data has proven such a challenge to date.

**Table 1.** Synthetic data results. (Shown are area-under-curve (AUC) values from receiver operating characteristic (ROC) curves for both outcome and biomarker predictions.)

run	covar.	outcome AUC	biomarker AUC
synth. (2 types)	SE	0.74 ± 0.04	0.59 ± 0.08
synth. (2 types)	Matern	0.70 ± 0.02	0.59 ± 0.04
synth. (2 types)	sum	0.70 ± 0.02	0.58 ± 0.04
synth. (2 types)	linear	0.71 ± 0.01	0.59 ± 0.10
synth. (1 type)	SE	0.68 ± 0.04	0.68 ± 0.04
synth. (1 type)	Matern	0.68 ± 0.04	0.62 ± 0.14
synth. (1 type)	sum	0.69 ± 0.04	0.62 ± 0.11
synth. (1 type)	linear	0.68 ± 0.04	0.66 ± 0.03

**Table 2.** Synthetic data ‘oracle’ results, where the correct features are known. (Shown are area-under-curve (AUC) values from ROC curves.)

run	covar.	outcome AUC
oracle (2 types)	linear	0.86 ± 0.02
oracle (2 types)	SE	0.87 ± 0.03
oracle (2 types)	Matern	0.86 ± 0.02
oracle (2 types)	sum	0.86 ± 0.03
oracle (1 type)	linear	0.78 ± 0.05
oracle (1 type)	SE	0.79 ± 0.04
oracle (1 type)	Matern	0.79 ± 0.04
oracle (1 type)	sum	0.79 ± 0.05

Table 3 shows comparison analyses using the Random Forest (RF) model [26], a stepwise logistic regression generalized linear model (GLM) method and a simple Support Vector Machine (SVM) with no feature selection. These represent well-known classification algorithms that would be sensible default choices for analysis of single data types such as those considered in this paper. The results show that all three comparison methods are significantly impacted by the presence of large numbers of noise features, under-performing in comparison to *FusionGP*. We also note that even in the oracle case, *FusionGP* shows somewhat superior performance.

**Table 3.** Synthetic data results for comparison methods. (For the ‘oracle’ runs, only the informative features are included in the analysis.)

data	method	prediction AUC	biomarker AUC
synth. (1 type)	RF	0.63 ± 0.03	0.57 ± 0.01
synth. (1 type)	stepwise GLM	0.61 ± 0.03	0.54 ± 0.01
synth. (1 type)	SVM (no selection)	0.66 ± 0.02	—
oracle (1 type)	RF	0.74 ± 0.01	—
oracle (1 type)	stepwise GLM	0.74 ± 0.01	—
oracle (1 type)	SVM (no selection)	0.75 ± 0.03	—

### 4.2 Results on METABRIC data

**Figure 2.**
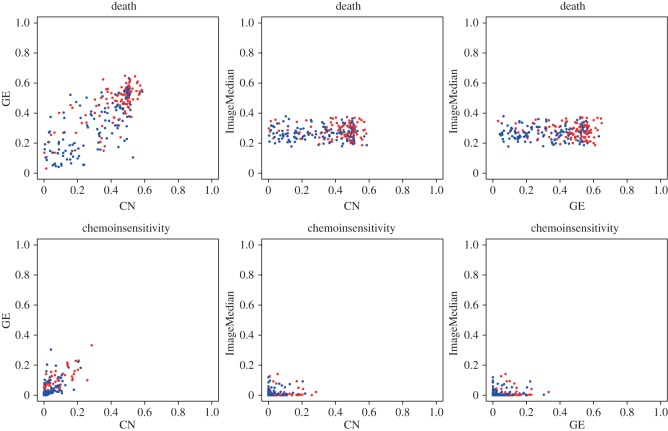
Scatter plots for outcome predictions of all patients, comparing results for single data types. Outcome, FALSE is shown in blue, outcome, TRUE is shown in red. CN, copy number; GE, gene expression.

We ran twofold cross-validation analyses to assess the performance of *FusionGP* on the METABRIC breast cancer data. For these analyses, we chose the SE covariance function, as it was best-performing on the synthetic data runs. Tables 4–6 show the prediction AUC from the cross-validation. We compared the AUC scores of *FusionGP* and each of the three comparison methods. The differences are computed for each single data type comparison in tables 4–6. Figure 2 shows scatter plots of the single data type results for *FusionGP*. Comparing the distributions of AUC scores for each method using the Wilcoxon rank sum test, we obtain *p*=0.66 (RF), 8×10^−4^ (stepwise GLM) and 4×10^−3^ (SVM). We therefore conclude that *FusionGP* significantly outperforms both stepwise GLM and SVM methods, and marginally outperforms RF for the METABRIC data types.

**Table 4.** ROC curve AUC results from analysing different combinations of METABRIC data types. (The best-performing method/s in each row are highlighted in bold. CN, copy number; GE, gene expression.)

	data	*FusionGP*	RF	GLM	SVM
chemoinsens.	CN	**0.72** ± **0.01**	0.68 ± 0.01	0.63 ± 0.05	0.61 ± 0.06
	GE	**0.78** ± **0.02**	0.74 ± 0.02	0.71 ± 0.01	0.70 ± 0.02
	image	**0.55** ± **0.04**	**0.58** ± **0.06**	**0.58** ± **0.01**	**0.57** ± **0.01**
	GE/CN	**0.78** ± **0.02**	—	—	—
	all	**0.78** ± **0.03**	—	—	—
death	CN	**0.69** ± **0.01**	0.68 ± 0.01	0.53 ± 0.02	0.62 ± 0.08
	GE	**0.75** ± **0.01**	0.74 ± 0.01	0.64 ± 0.05	0.68 ± 0.03
	image	**0.60** ± **0.01**	**0.59** ± **0.01**	0.49 ± 0.02	**0.60** ± **0.01**
	GE/CN	**0.75** ± **0.01**	—	—	—
	all	**0.75** ± **0.01**	—	—	—

**Table 5.** ROC curve AUC results, using only the 119 ER− patients. (The best-performing method/s in each row are highlighted in bold.)

	data	*FusionGP*	RF	GLM	SVM
chemoinsens.	CN	**0.59** ± **0.10**	**0.60** ± **0.14**	0.52 ± 0.07	0.54 ± 0.16
	GE	**0.59** ± **0.07**	**0.60** ± **0.02**	0.51 ± 0.05	0.55 ± 0.02
	image	**0.61** ± **0.07**	**0.60** ± **0.03**	0.55 ± 0.01	**0.59** ± **0.10**
	GE/CN	**0.59** ± **0.11**	—	—	—
	all	**0.59** ± **0.08**	—	—	—
death	CN	**0.56** ± **0.07**	**0.54** ± **0.06**	0.51 ± 0.02	0.46 ± 0.01
	GE	**0.55** ± **0.03**	**0.56** ± **0.06**	**0.51** ± **0.06**	**0.52** ± **0.04**
	image	**0.72** ± **0.03**	**0.73** ± **0.04**	0.69 ± 0.02	0.69 ± 0.01
	GE/CN	**0.55** ± **0.02**	—	—	—
	all	**0.67** ± **0.01**	—	—	—

**Table 6.** ROC curve AUC results, using only the 345 ER+ patients. (The best-performing method/s in each row are highlighted in bold.)

	data	*FusionGP*	RF	GLM	SVM
chemoinsens.	CN	**0.68** ± **0.01**	0.62 ± 0.01	0.58 ± 0.03	0.62 ± 0.02
	GE	**0.70** ± **0.02**	0.66 ± 0.03	**0.64** ± **0.11**	0.60 ± 0.01
	image	0.52 ± **0.04**	**0.58** ± **0.08**	0.46 ± 0.08	0.50 ± 0.09
	GE/CN	**0.70** ± **0.02**	—	—	—
	all	**0.70** ± **0.03**	—	—	—
death	CN	**0.71** ± **0.02**	**0.71** ± **0.03**	0.58 ± 0.11	0.60 ± 0.03
	GE	**0.75** ± **0.01**	0.74 ± 0.01	**0.75** ± **0.01**	0.68 ± 0.01
	image	**0.62** ± **0.04**	**0.64** ± **0.04**	0.52 ± 0.04	**0.60** ± **0.05**
	GE/CN	**0.75** ± **0.01**	—	—	—
	all	**0.75** ± **0.01**	—	—	—

For analysis using death as outcome, we found a striking difference between the ER− and ER+ item subsets. For the ER+ items, the molecular data are highly informative. However, the combination of copy number and gene expression data produce results consistent with those obtained for gene expression alone. We suggest that this is due to a duplication of information in that the effect of the gene expression accompany copy number alteration. In ER− tumours both molecular data types are poorly informative. The image features, however, are highly informative, suggesting that cell morphology information are important in predicting survival in breast cancer.

For predicting chemoinsensitivity, we found that for the ER+ samples the molecular data are most informative. For the ER− samples, our results suggest all three data types contain some relevant information. Of note for chemoinsensitivity is the performance of the all-samples case. The predictive performance for this case is significantly better than that of either ER− or ER+ cases. There must, therefore, be significant information sharing between the ER− and ER+ cases, in contrast with the analyses for death, where the two ER subsets are highly distinct.

We compared *FusionGP* for single data types to the RF, stepwise GLM (logistic regression) and SVM with no feature selection. Of the outcome/single-data-type combinations considered in tables 4–6, *FusionGP* is either the best performing method or within one standard deviation of the best result in 26 of the 27 cases. RF also performed well, with joint-best performance in 16 cases and overall best performance in one case. Stepwise GLM and the SVM were generally outperformed by both *FusionGP* and RF, with a few exceptions.

For the image features, we found that across all analyses about a third of them were selected. This suggests that there is duplicate information, which is expected due to the nature of their construction. For example, acircularity (median acirc) and HuÃŢs first moment or I1 (median I1) both measure shape irregularity. There is minimal downside here in terms of predictive performance, although it suggests that it may be possible to construct a smaller set of equally informative image features, which would have some benefit in terms of the run time of any analysis.

**Figure 3.**
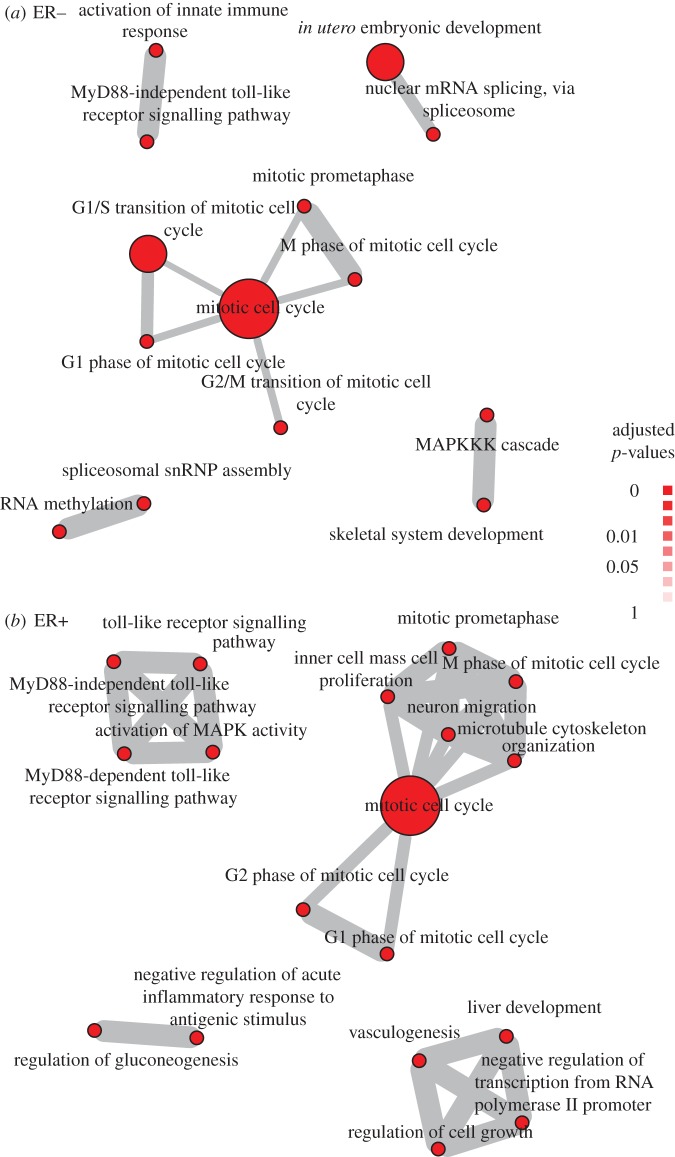
Enrichment maps for *chemoinsensitivity* as outcome for (*a*) ER− and (*b*) ER+ tumours. Shown are biological process that are statistically over-represented in the copy number features for the single data type *FusionGP* analysis, as determined by a hypergeometric test (*p*<1^−4^). The size of the red circles indicates the number of genes with a given pathway; the grey lines show where the same gene is shared across a pair of pathways, with line thickness indicating the number of genes. Enrichment maps were generated using the R package *HTSanalyzeR*. Singleton/unconnected nodes are not shown.

We analysed the most strongly selected features for each data type. For the molecular types, we looked for enrichment in gene ontology (GO) ontologies and KEGG pathways. We focused on biological process in GO to identify processes underlying features predicted to be informative for important clinical phenomenon. Figure 3 shows specific biological processes enriched in copy number features that were found to be predictive of chemoinsensitivity by our algorithm in ER− and ER+ tumours. Multiple cell cycle processes forming large cell cycle modules are evident in both ER− and ER+ tumours, signifying the role of cell-cycle pathways across ER status yet highlighting different cell-cycle phases for different ER status, e.g. transition phases including G2/M and G1/S for ER− and G1 and G2 for ER+. Notably, activation of innate immune response is significantly enriched in the selected features for predicting chemoinsensitivity only in ER− tumours. This is consistent with recent evidences showing the importance of immune response in achieving pathological complete response to chemotherapy in ER− tumours [27,28]. Our result again confirmed their observations yet extended to the genomics level, indicating specific genomic aberrations that could underly innate immune response and influence the response of ER− patients to chemotherapy.

**Figure 4.**
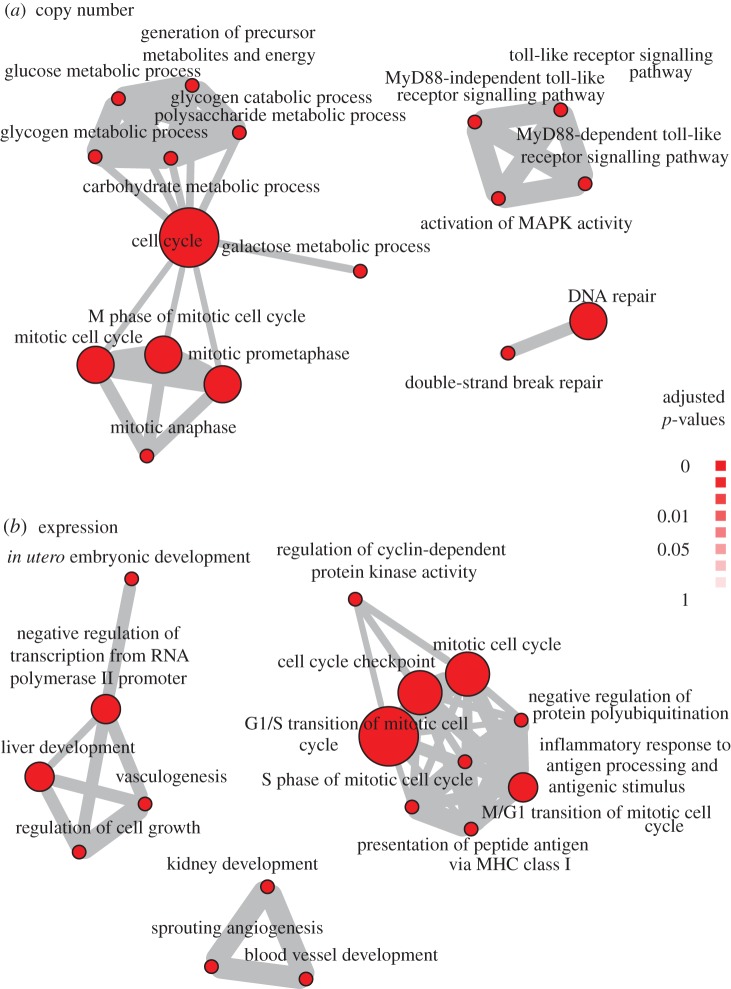
Enrichment maps for *deaths* as outcome for (*a*) copy number and (*b*) expression features selected for ER+ tumours. Shown are biological process that are statistically over-represented in the corresponding feature types for the single data type *FusionGP* analysis, as determined by a hypergeometric test (*p*<1^−4^).

We examined copy number and gene expression features for predicting deaths in ER+ because of their good performance as indicated in table 6. Compared with figure 3, large cell-cycle modules can also be found in the biological processes enriched in both feature types (figure 4). Of particular note, however, is the DNA repair processes enriched only in the copy number features. It is well known that BRCA1, a key mediator of the DNA repair pathways, remain one of the most important genes for breast cancer. Specifically for ER+ patients, loss of BRCA1-mediated transcriptional activation of ER expression can lead to increased resistance to ER antagonists [29]. Thus, the presence of genomic features involved in DNA repair when predicting deaths in ER+ patients support the accuracy of our algorithm in searching for biologically meaningful features.

Gene expression features useful for predicting ER+ deaths include those involved in angiogenesis (figure 4*b*). This indicates that microenvironmental changes such as angiogenesis, which can be captured by gene expression data generated from our whole-tumour material, can be found in patients with poor prognosis. Angiogenesis is a known hallmark of poor prognosis in breast cancer as supported by multiple lines of evidences [30]. Meanwhile, ER is known to be capable of mediating angiogenesis, but its mechanism remains elusive Losordo & Isner [31], and our result here not only supports this but also reveals specific genes from our unsupervised analysis that may help define the regulatory mechanism. Taken together, results from our proposed model are supported by current knowledge of important genomic aberrations and microenvironmental features implied in insensitivity to chemotherapy and poor prognosis, in addition to identifying new features that can potentially help define mechanisms of these critical events.

## 5. Conclusion

We have presented *FusionGP*, a non-parametric Bayesian method for combining multiple data types to predict binary clinical outcomes. *FusionGP* generalizes the notion of feature selection for biomarker discovery, allowing for simultaneous, sparse feature selection across multiple heterogeneous data types.

Results on synthetic data show that *FusionGP* is effective at making predictions using noisy, sparse data and that it can identify the informative features. Combining two synthetic data types leads to superior predictive results, and also that *FusionGP* outperforms the RFs classifier, stepwise logistic regression and also a simple SVM. We suggest that this is because these standard methods do not explicitly account for the sparse nature of the synthetic data.

We present a range of analyses of the METABRIC breast cancer dataset, including gene expression, copy number alterations and H&E image-derived morphological feature data, and considering as outcomes *dea*th and *chemoinsensitivity*. We note that because the results are for a single collection of datasets, and using cross-validation rather than an independent test set, some caution in the interpretation of these results is warranted. With this in mind, from these analyses we draw a number of conclusions.

— *FusionGP* consistently outperforms both stepwise GLM and SVM methods, and marginally outperforms RF for the METABRIC data types.
— For prediction of disease-specific death, the molecular data are highly informative for the ER+ tumours, whereas for the ER− tumours the image features significantly outperform the molecular data.
— The enriched biological processes for the ER+ cases highlight specific genomic alterations in the DNA repair pathway, which is indicative of DNA repair-mediated ER expression activation and imply an influence on response to ER treatment.
— For prediction of chemoinsensitivity, ER− and ER+ tumours share common cell-cycle-related processes in the feature selected, yet differs in processes signifying the role of immune infiltration in the microenvironment in response to chemotherapy in ER− tumours.
— We therefore conclude that it is important to account for both the ER subtypes and also common underlying structure that is shared by all tumour samples.

We note that in practical terms, complex methods such as *FusionGP* have much longer run times than many of the equivalent standard methods. For example, if one is interested mainly in making predictions using the METABRIC data, one can achieve only marginally worse results using the much-quicker RF algorithm. *FusionGP* does also give a useful ability to better explain the biology underlying the data, as we have done in this paper. Nevertheless, we note that improvements to the run time of *FusionGP* would greatly improve its utility.

Given these results, we believe a number of interesting future directions present themselves. The image features, and to some extent the molecular features, contain significant redundancy. Developing models that learn a new set of (uncorrelated) latent features, rather than simply selecting the existing features, may allow us to refine our biological insights. It would also be interesting to encode more complex structures between the different data types—for example, we know that copy number alterations will effect gene expression patterns. Previous experience suggests that modelling such structure can add significant complexity to the modelling process, but it is certainly worthy of further investigation. Finally, fast approximations for the *FusionGP* algorithm will become increasingly valuable as multi-type cancer datasets becoming increasingly large.

We live in an exciting time for cancer research, where a range of new measurement technologies are giving us unprecedented access to the inner workings of tumours and cancer cells. Developing new methods to better use this data is vital if we are to make the most of this wave of new data.

## Data accessibility

All data have been deposited at the European Genome-Phenome Archive hosted by the European Bioinformatics Institute, under accession number EGAS00000000083. They can also be accessed upon request via the METABRIC data access committee (metabric@cruk.cam.ac.uk). The *FusionGP* Matlab code and synthetic data can be downloaded from https://sites.google.com/site/fusiongppaper/.
